# Evaluation of an I177L gene-based five-gene-deleted African swine fever virus as a live attenuated vaccine in pigs

**DOI:** 10.1080/22221751.2022.2148560

**Published:** 2022-12-18

**Authors:** Yingnan Liu, Zhenhua Xie, Yao Li, Yingying Song, Dongdong Di, Jingyi Liu, Lang Gong, Zongyan Chen, Jinxian Wu, Zhengqin Ye, Jianqi Liu, Wanqi Yu, Lu Lv, Qiuping Zhong, Chuanwen Tian, Qingqing Song, Heng Wang, Hongjun Chen

**Affiliations:** a Shanghai Veterinary Research Institute, CAAS, Shanghai, People’s Republic of China; b Biosafety Research Center, CAAS, Shanghai, People’s Republic of China; c The Spirit Jinyu Biological Pharmaceutical Co. Ltd, Hohhot, People’s Republic of China; d South China Agricultural University, Guangdong, People’s Republic of China

**Keywords:** African swine fever virus, gene-deleted, live attenuated vaccine, safety, protective efficacy

## Abstract

African swine fever (ASF) is a highly contagious disease of domestic and wild pigs caused by the African swine fever virus (ASFV). The current research on ASF vaccines focuses on the development of naturally attenuated, isolated, or genetically engineered live viruses that have been demonstrated to produce reliable immunity. As a result, a genetically engineered virus containing five genes deletion was synthesized based on ASFV Chinese strain GZ201801, named ASFV-GZΔI177LΔCD2vΔMGF. The five-gene-deleted ASFV was safe and fully attenuated in pigs and provides reliable protection against the parental ASFV strain challenge. This indicates that the five-gene-deleted ASFV is a potential candidate for a live attenuated vaccine that could control the spread of ASFV.

## Introduction

The African swine fever virus (ASFV) causes a highly contagious and lethal disease in domestic pigs and wild boars, with mortality rates approaching 100% [1–3]. The pig industry is considered to be at risk worldwide due to this disease. ASFV belongs to the only member of the genus *Asfivirus* within the *Asfarviridae* family [3]. Infection with the virus is mainly caused by direct contact with the mouth or nose, and it has multiple modes of transmission. The majority of it is found in blood, tissue fluid, internal organs, secretions, and excreta.

A commercial vaccine is not yet available for the prevention or treatment of this disease. African swine fever outbreaks are still controlled primarily by quarantine and slaughter. Different vaccine strategies have been attempted to develop a safe and effective ASF vaccine in the past decades [4–6], but it seems that most of the candidates were still in the laboratory. It has been shown that pigs are protected from infection from the homologous strains following vaccination with several naturally attenuated isolates and genetically engineered isolates or viruses attenuated by a passage in tissue culture [7–14]. Due to the complexity of ASFV and biosecurity concerns, these vaccine candidates haven't been approved yet. Recently, gene-deleted strain ASFV-GZ-ΔI177L has shown potential as live attenuated ASF vaccine [10], but it still has a low level of virus shedding in nasal [15], which is a huge challenge to long-term biosecurity prevention and control. With this knowledge, perhaps, the safety profile of ASFV-GZ-ΔI177L needs to be further improved. According to the experiences and understanding of previous studies, gene-deleted attenuated ASFVs are still the most effective vaccine candidates to prevent ASF, but a carefully chosen target gene is crucial, often it is necessary to consider the safety of immunization, while also considering the suitability of protection, to achieve a balance between safety and efficacy.

Our study reports that a five-gene-deleted ASFV based on the *I177L* gene is fully attenuated in pigs, cannot convert to virulent strains and confers immunity against challenges with virulent parental viruses. The five-gene-deleted ASFV showed a good balance between safety and immunogenicity and could potentially be a new live, attenuated vaccine candidate for ASF control.

## Results

### Generation of the gene-deleted ASFVs

**Figure 1.**
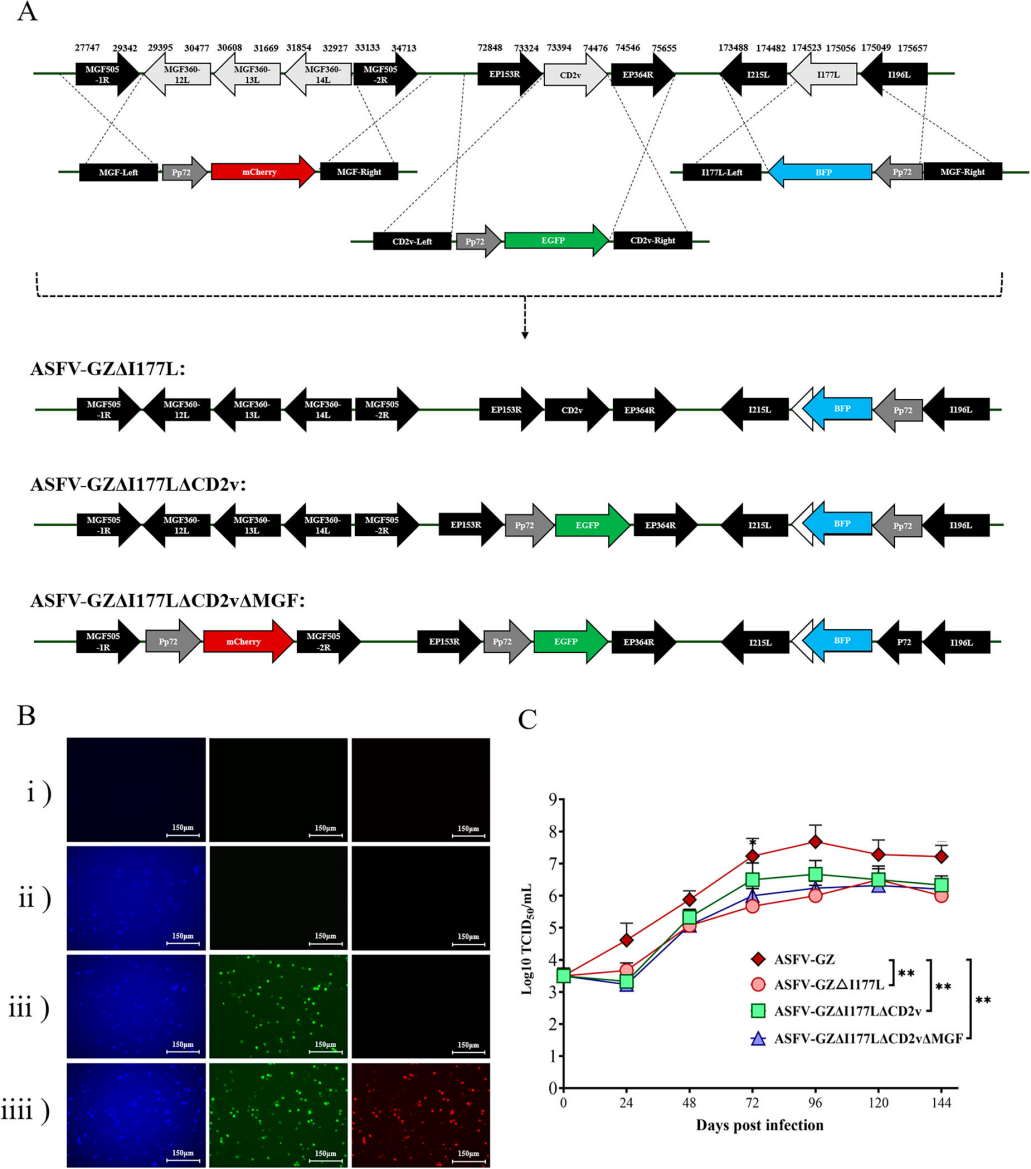
*Illustrating generation of different gene-deleted African swine fever viruses:* (A) Schematic for the construction of different gene-deleted ASFVs. The deleted gene segments were replaced by the Pp72-EGFP, Pp72-mCherry, or Pp72-BFP expression cassette as shown. (Note: The white arrowhead to the left of the BFP represent remaining 3'-*I11*7L gene sequence). (B) The virus-infected primary BMDM cells expressing differential fluorescence. (C) *In* vitro growth characteristics of different gene-deleted ASFVs where primary BMDM cells are infected (0.01 m.o.i) with the viruses, and virus yields were titrated at the indicated times post-infection. Data are represented as means and standard deviations. The viral titers were exhibited by log_10_ TCID_50_/ml. (**P <* *0.01, ***P <* *0.001).

Three gene-deleted ASFVs including ASFV-GZΔI177L, ASFV-GZΔI177LΔCD2v, and ASFV-GZΔI177LΔCD2vΔMGF were constructed by targeting *I177L* gene, a combination of *I177L* and *EP402R* genes, and combination of *I177L*, CD2v and ASF virulence-associate *MGF360-12L*–*MGF360-14L* gene clusters [13,16], respectively, using DNA homologous recombination technique [17–19] (Figure 1(A)). The gene-deleted ASFVs were purified through successive rounds of plaque purification and limiting dilution, followed by PCR analysis to verify the absence of the parental ASFV-GZ virus and the desired deletion (Figure 1(B)). We compared the growth kinetics of these three gene-deleted ASFVs to that of ASFV-GZ at different time points on primary BMDM cells. The results showed that these three gene-deleted ASFVs displayed similar growth patterns without significant differences. However, compared to the parental strain, lower replication levels were observed for these three gene-deleted ASFVs from 24 h post-infection, which were 17.7 – fold (ASFV-GZ/ASFV-GZΔI177L), 19.41 – fold (ASFV-GZ/ASFV-GZΔI177LΔCD2v) and 24.21 – fold (ASFV-GZ/ASFV-GZΔI177LΔCD2v ΔMGF) lower than that of the parental ASFV-GZ virus, respectively (*p *< 0.001). And the differences gradually narrowed to within 15 – fold as the infection time increased (48–120 hpi, *P* < 0.05) (Figure 1(C)). These findings suggested that deletion of these genes significantly decreased viral replication *in vitro*.

### Evaluation of virulence in pigs

**Figure 2.**
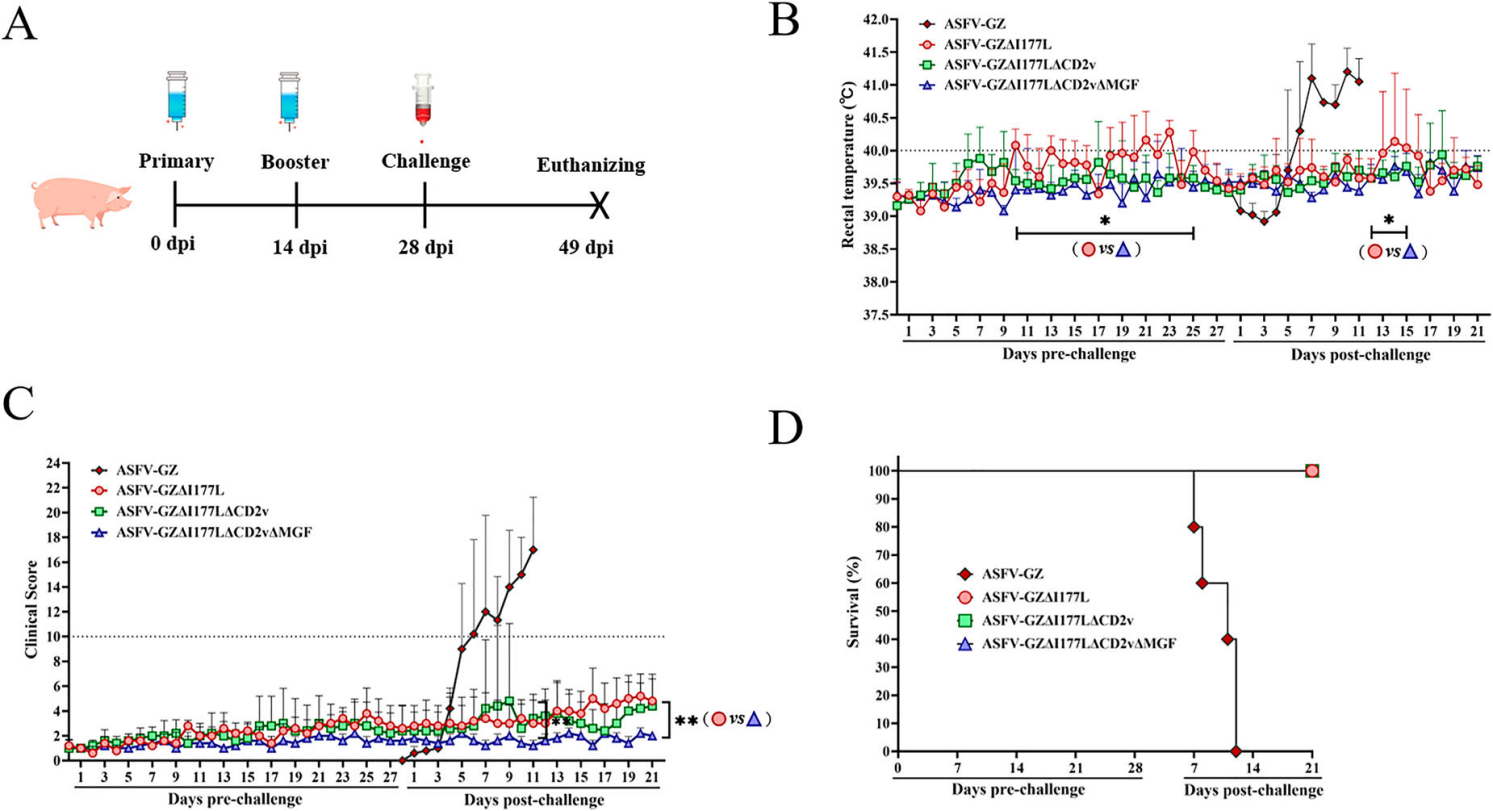
*In vivo evaluation of different gene-deleted African swine fever viruses (ASFVs).* (A) Timeline for animal experiments. The groups of commercial pigs were inoculated twice with 10^5.00^ TCID_50_ of different gene-deleted ASFVs and then challenged at 28 dpi with lethal ASFV-GZ virus inoculated intramuscularly (i.m.). Pigs were observed for 21 days post-challenge and then euthanized. (B) The rectal temperature of the pigs inoculated with different gene-deleted ASFVs and challenged with lethal ASFV-GZ virus. *P < 0.05, the mean rectal temperature of pigs in the ASFV-GZΔI177L group was significantly higher than that in the ASFV-GZΔI177LΔCD2vΔMGF group at 10–23 days post-inoculation and 13–16 days post-challenge. (C) The clinical score of the pigs were recorded and calculated after inoculation and challenge. **P < 0.01, the mean clinical score in the ASFV-GZΔI177L group was significantly higher than that in the ASFV-GZΔI177LΔCD2vΔMGF group. (D) Survival curve of the pigs after inoculation and challenge.

**Figure 3.**
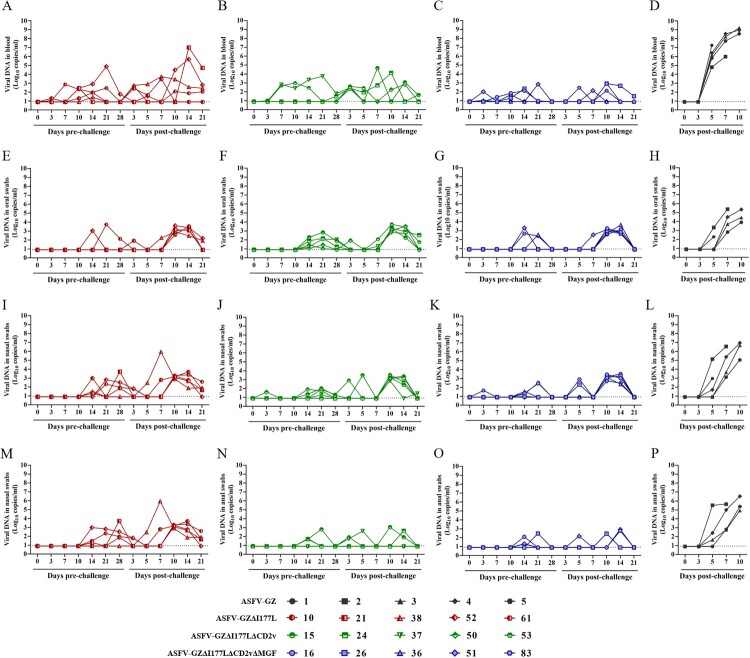
*Evaluation of virulence protective efficacy in pigs.* (A–D) The viremia in pigs after inoculation and challenge. (E–H) The virus shedding in oral-swabs from pigs after inoculation and challenge. (I–L) The virus shedding in nasal swabs from pigs after inoculation and challenge. (M–P) The virus shedding in anal swabs from pigs after inoculation and challenge.

To determine whether these gene-deleted ASFVs were attenuated in pigs, domestic piglets about 15–30 kg were inoculated intramuscularly (IM) with 10^5.00^ TCID_50_ of either the gene-deleted viruses (Group ASFV-GZΔI177L, ASFV-GZΔI177LΔCD2v, and ASFV-GZΔI177LΔCD2vΔMGF, *n* = 5/group) or parental ASFV GZ201801 strain (Group ASFV-GZ, *n* = 5) and boosted at 14 dpi and observed the pigs for two more weeks (Figure 2(A)). The animals were monitored for rectal temperature and other clinical symptoms and scored using the clinical scoring system as described previously [20]. All the animals inoculated with these three gene-deleted ASFVs survived the immunization observation period (28 days) before the virus challenge. The variations in rectal temperature and the clinical score of each group were recorded and shown in Figure 2(B,C). Two of five animals inoculated with ASFV-GZΔI177L develop transient low-grade fever (temperature, < 41°C) at 10–11 dpi and 18–25 dpi, and then the temperature returned to normal. As the rectal temperatures were relatively stable, the two animals (pigs, No. 52 and No. 61) showed mild joint swelling after 20–21 days post-inoculation. All the animals inoculated with ASFV-GZΔI177LΔCD2v or ASFV-GZΔI177LΔCD2vΔMGF remained healthy and survived for the whole immunization observation period (28 days). The viremia of each group was recorded in Figure 3(A–C). A low level of viremia was detected from the 3rd day and then maintained at a low mean viremia level (< 10^3.00^ copies/ml). Noticeably, there were no viral DNAs detected in any of the animals inoculated with ASFV-GZΔI177LΔCD2vΔMGF at 28 dpi, indicating that the immunized virus (10^5.00^ TCID_50_/Dose) can be completely expunged during the 28-day immunization period (Figure 3(A–D)). These results indicated that ASFV-GZΔI177LΔCD2vΔMGF was completely loss virulence to domestic pigs, and was more friendly to domestic pigs compared to ASFV-GZΔI177L and ASFV-GZΔI177LΔCD2v.

### Exploration of protective efficacy in pigs

To investigate whether the attenuated ASFVs would induce protective immunity, the animals pre-inoculated with these three gene-deleted ASFVs were challenged with 10^4.00^ TCID_50_ of ASFV-GZ virus. An additional group of unvaccinated pigs (*n* = 5) was included as a mock-vaccinated group and challenged under the same conditions. Following the virus challenge, the animals were monitored for rectal temperature and other clinical symptoms and scored as described previously [19]. The mock-vaccinated pigs inoculated with ASFV-GZ exhibited an increased rectal temperature (> 40 °C) at 3–5 dpc, followed by ASF-related clinical symptoms, including depression, inappetence, or joint swelling. All the pigs died within 12 d-post-challenge (dpc) (Figure 2(B–D) and Table 1). Conversely, all pigs pre-inoculated with these three gene-deleted ASFVs survived the challenge (Figure 2(D)). However, in the ASFV-GZΔI177L-inoculated group, both pigs with mild joint swelling developed transient fever since 13–14 dpc and last 3–4 days of post-challenge, followed by the rectal temperature returned to normal, but joint swelling gradually worsened. The other three animals were clinically normal throughout the challenge observation period. In the ASFV-GZΔI177LΔCD2v-inoculated group, one developed fever for only two days before rapidly recovering (No. 37 pig, 17–18 dpc, 40.9–41.1°C). However, four of the five animals showed joint swelling after ASFV-GZ challenge at 11–15 days post-challenge. All five pigs in the ASFV-GZΔI177LΔCD2vΔMGF-inoculated group did not present any clinical signs related to ASF following the challenge. The viremia was detected and the results are shown in Figure 3(A–D). It was shown that, after the challenge, high levels of viremia were detected in the challenge control group, and all three gene-deleted ASFV groups showed lower levels of viremia, this was probably caused by the challenge virus ASFV-GZ. Noticeably, viremia in the ASFV-GZΔI177LΔCD2vΔMGF vaccinated pigs was barely detectable at days 28 after inoculation (below the detection limit of 10^0.94^ copies/ml) (Figure 3(C)).

**Table 1.** Swine survival and fever response of the pigs following challenge with the lethal ASFV-GZ virus.

Viruses	No. of survivors	Time to death (Mean [SD]) (days)	Data for fever		
			No. of days to onset (mean [SD])	Duration (days) (mean [SD])	Maximum daily temp (°C)
ASFV-GZΔI177L	5/5	/	9.3 [3.51]	3.5 [0.71]	41.7
ASFV-GZΔI177LΔCD2v	5/5	/	10 [0]	2 [0]	40.2
ASFV-GZΔI177LΔCD2vΔMGF	5/5	/	/	/	40.0
ASFV-GZ	0/5	10 [2.35]	5.8 [1.30]	4.2 [1.30]	41.6

There was evidence of viral shedding in oral swabs (Figure 3(E–H)), nasal swabs (Figure 3(I–L)), and anal swabs (Figure 3(M–P)), respectively. In ASFV-GZΔI177L and ASFV-GZΔI177LΔCD2v-inoculated groups, lower levels of viral DNA copies were detected from all swabs at 14 dpi and maintained until 28 dpi, compared to the challenge control group. The results of oral, nasal, and anal swabs showed consistent results of qPCR. However, no viral shedding was detected during the immunization observation period in the ASFV-GZΔI177LΔCD2vΔMGF-inoculated group. After the challenge, virus shedding was detected from 7 dpc with low viral DNA copies and peaked at 10–14 dpc (around 10^3.50^ copies/ml), then drastically decreased by 21 dpc in all animals of these three gene-deleted ASFVs groups. Importantly, all pigs in the ASFV-GZΔI177LΔCD2vΔMGF-inoculated group were not able to detect the virus shedding 21 days post-challenge.

**Figure 4.**
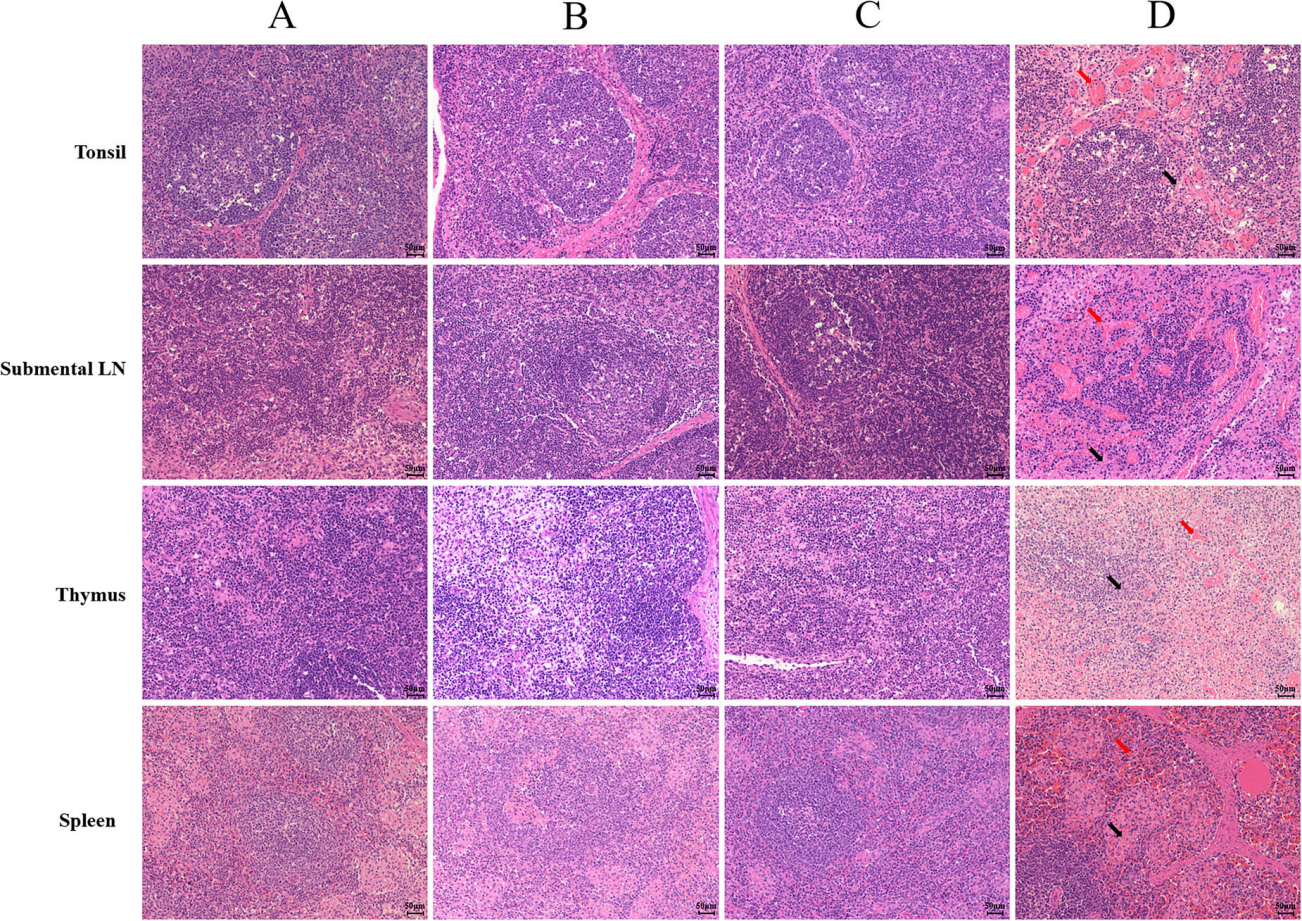
*The histopathological sections (H&E) of the tonsil, submental lymph node, thymus, and spleen.* The organs and tissues were collected from the pigs inoculated with 10^5.00^ TCID_50_ of ASFV-GZΔI177L (Column **A**), ASFV-GZΔI177LΔCD2v (Column **B**), and ASFV-GZΔI177LΔCD2vΔMGF (Column **C**) and then challenged with 10^4.00^ TCID_50_ of ASFV-GZ. Column **D**, the tissues were collected from the mock pigs challenged with 10^4.00^ TCID_50_ of ASFV-GZ. Notes: Submental LN–Submental lymph node.

In addition to the dead pigs, necropsies were conducted on the challenge survivors at 21 dpc. The dead pigs in the ASFV-GZ group presented with typical gross changes of ASF, such as visceral haemorrhage and swollen or necrotic lymph nodes, but the survivors in these three gene-deleted ASFV virus immunized groups exhibited no pathological changes. Major immune-related organs or tissues, including the spleen, thymus, tonsil, and submaxillary nodes, were histopathologically examined. In all dead pigs, haemorrhagic necrosis (red arrows) and apoptosis (black arrows) of lymphocytes were evident, whereas the surviving pigs inoculated with each of these three gene-deleted viruses did not show demonstrated any changes (Figure 4). As a result, all three gene-deleted viruses could fully protect pigs against lethal ASFV-GZ virus challenge in this period.

### Evaluation of host immune response

To examine the immune responses of pigs to these three gene-deleted ASFVs, sera of experimented animals were sampled at 3, 7, 10, 14, 21, and 28 dpi for detection of cytokine and anti-ASFV antibody levels.

**Figure 5.**
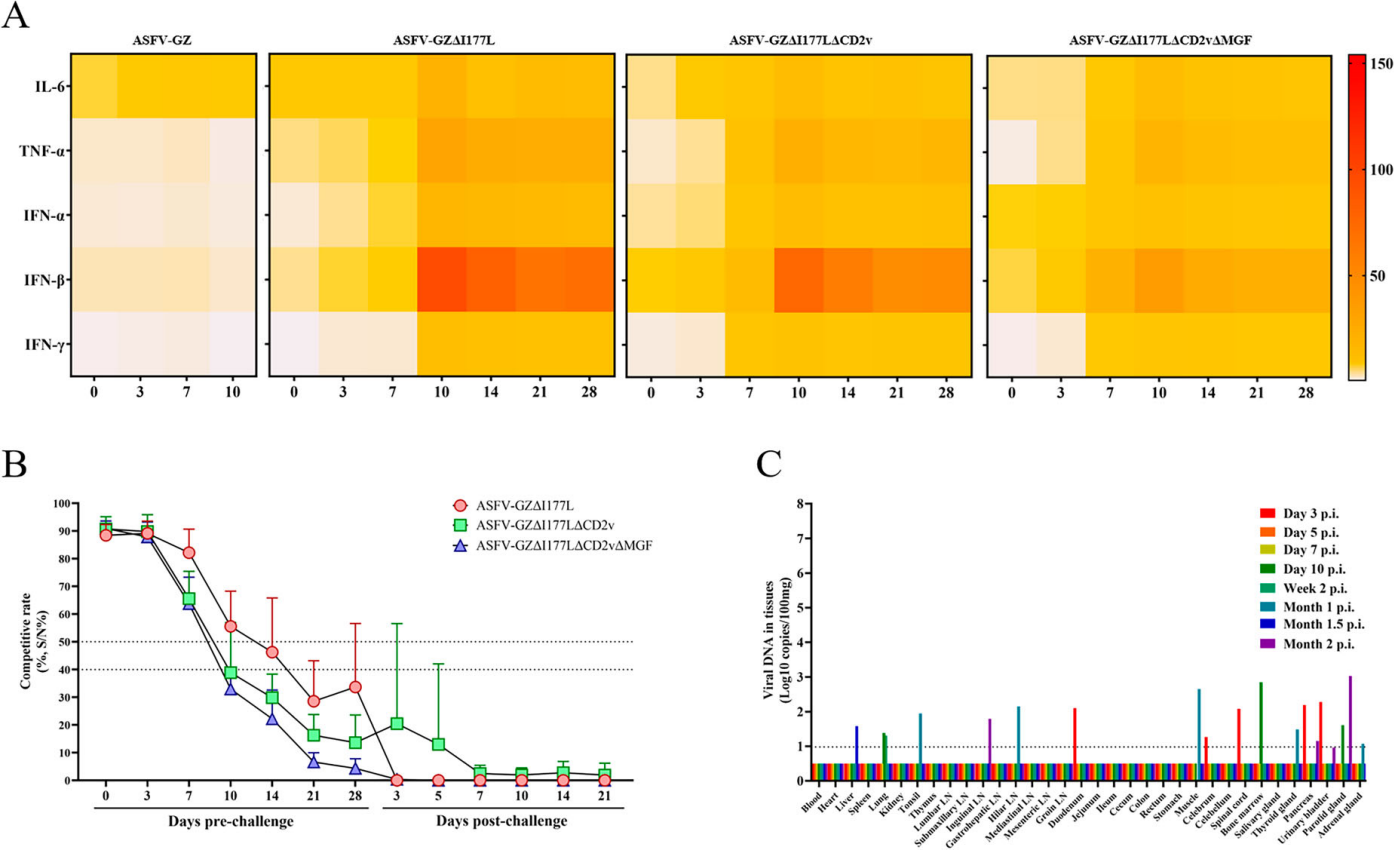
*Evaluation of host immune response.* (A) Assessment of cytokines in pigs inoculated with ASFV-GZ, ASFV-GZΔI177L, ASFV-GZΔI177LΔCD2v, or ASFV-GZΔI177LΔCD2vΔMGF. The heatmap was drawn using the mean of each group. (B) The antibody response curves from different groups of pigs after inoculation and challenge. The anti-ASFV antibodies have been detected using an ASF competitive ELISA antibody detection kit. The competitive percentage (S/N %) was calculated. Samples presenting a S/N %: ≤ 40% were considered positive; 40–50% was considered doubtful; ≥ 50% was considered negative. (C) Replication of ASFV-GZΔI177LΔCD2vΔMGF in pigs. 8 two-month-old commercial domestic pigs were inoculated with a dose of 10^7.00^ TCID_50_ of ASFV-GZΔI177LΔCD2vΔMGF virus and one pig at indicated time points was euthanized to collect blood, oral swabs, nasal swabs, anal swabs and organs for viral DNA detection.

The cytokine kinetic properties of pigs inoculated with these three gene-deleted ASFVs were evaluated and compared with those of pigs infected with the ASFV-GZ strain. The expression levels of IL-6, TNF-α, IFN-α, IFN-β and IFN-γ in the sera of pigs of each group were detected via ELISA during the immunization (Figure 5(A)). The results showed that pigs infected with ASFV-GZ had slight or negligible expression of cytokines. However, pigs inoculated with these three gene-deleted ASFVs all showed an increase in cytokine induction at 7 dpi and peaked at 7–10 dpi, followed by a reduction until 28 dpi. Notably, both IFN-β and TNF-α exhibited extremely high expression levels in the ASFV-GZΔI177L-inoculated (69.73–94.66 ng/ml) and ASFV-GZΔI177LΔCD2v-inoculated groups (47.46–75.27 ng/ml), compared to slightly lower levels in the ASFV-GZΔI177LΔCD2vΔMGF group (21.37–35.20 ng/ml). These results suggest that these three gene-deleted ASFVs are highly capable of inducing cytokine expression.

Serum levels specific anti-ASFV antibodies were evaluated by ELISA in these three gene-deleted ASFVs groups during the immunization and challenge period. Detection of antibody levels began at 14 days post-inoculation, peaked at 21 days post-inoculation, and remained constant for 28 days post-inoculation. In contrast, the anti-ASFV antibody induced by ASFV-GZΔI177LΔCD2vΔMGF seems to be faster and higher levels than that of ASFV-GZΔI177L and ASFV-GZΔI177LΔCD2v. A slight increase in antibodies level was observed after the challenge (Figure 5(B)). Subsequently, the neutralization test of the sera collected at 28 dpi in the three groups were performed to evaluate the neutralizing antibody levels. However, no viral neutralizing activity was observed in all the sera.

### Replication evaluating in pigs

To investigate the replication of ASFV-GZΔI177LΔCD2vΔMGF in pigs, 8 two-month-old commercial domestic pigs were inoculated with a dose of 10^7.00^ TCID_50_ of the virus, and one pig at indicated time points was euthanized to collect blood, oral swabs, nasal swabs, anal swabs and organs for detection of viral DNA. As shown in Figure 5(C), viral DNA was detected in the duodenum, cerebrum, spinal cord, pancreas, and urinary bladder of the pigs that were euthanized on 3 dpi (10^1.27^–10^2.28^ copies/ml), in the lung, bone marrow, and parotid gland on 10 dpi (10^1.16^–10^2.85^ copies/ml), in the lung on 14 dpi (10^1.31^ copies/ml), in tonsil, hilar LN, muscle, thyroid gland and adrenal gland on 30 dpi (10^1.07^–10^2.65^ copies/ml), and liver on 45 dpi (10^1.58^ copies/ml). These results indicated that the virus replication and distribution could be detected from day 3 p.i. in pigs inoculated with an overdose (10^7.00^ TCID_50_/Dose) of ASFV-GZΔI177LΔCD2vΔMGF virus.

### Safety evaluation in pigs

**Figure 6.**
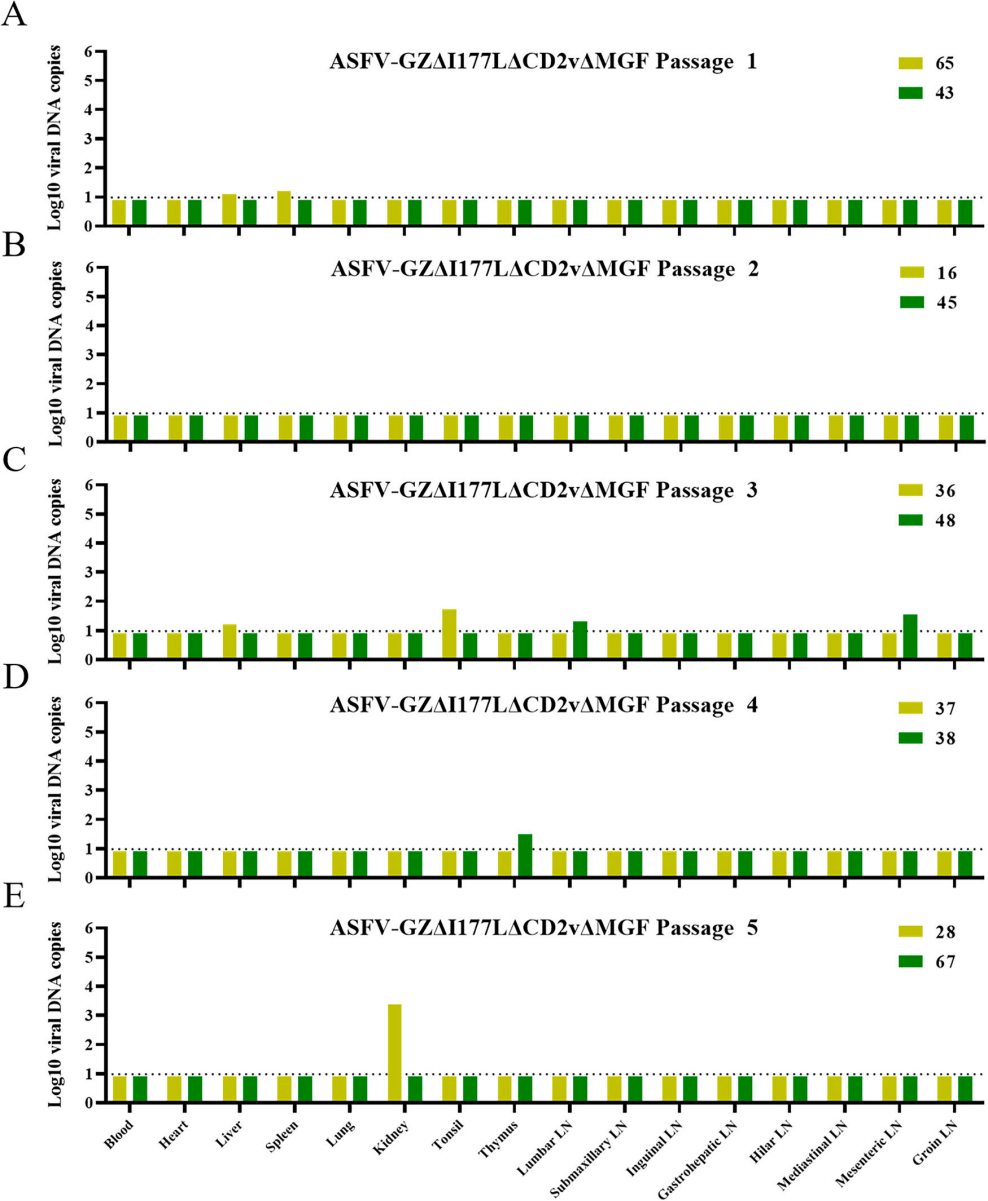
*Safety evaluation in pigs.* The ASFV-GZΔI177LΔCD2vΔMGF virus was serially passaged in pigs, indicating samples collected from the pigs that were euthanized on 3 dpi for viral DNA detection. The viral DNA copies of ASFV-GZΔI177LΔCD2vΔMGF virus from the 1st to 5^th^ passage are shown in panels A to E, respectively. Note: LN–lymph node.

We then sought to determine whether the attenuated strain of ASFV-GZΔI177LΔCD2vΔMGF could convert into a virulent strain when replicated in susceptible animals. The virus was serially passaged in pigs for five passages. Groups of three commercial piglets were intramuscularly inoculated with 10^7.00^ TCID_50_ of ASFV-GZΔI177LΔCD2vΔMGF virus, two of them were euthanized at 3 dpi, and the rest one was observed for 21 days. The organs of these pigs were collected for viral DNA detection as described above. In the ASFV-GZΔI177LΔCD2vΔMGF-inoculated group, viral loadings in the liver and spleen were very limit below the detection limit of 10^0.94^ copies/ml on 3 dpi. The DNA-positive samples were homogenized and passaged four more times in pigs. We found that viral DNA could not be detected in the blood, but was detected in individual tissues or organs, and the viral load is maintained at a low level (< 10^3.38^ copies/ml) (Figure 6). Importantly, the pigs inoculated with the passage 5 virus did not present any ASF-related clinical signs. These results indicated that the ASFV-GZΔI177LΔCD2vΔMGF is unlikely to convert to virulent strain during its replication in pigs.

### *In vitro* and *in vivo* gene stability assessment

**Figure 7.**
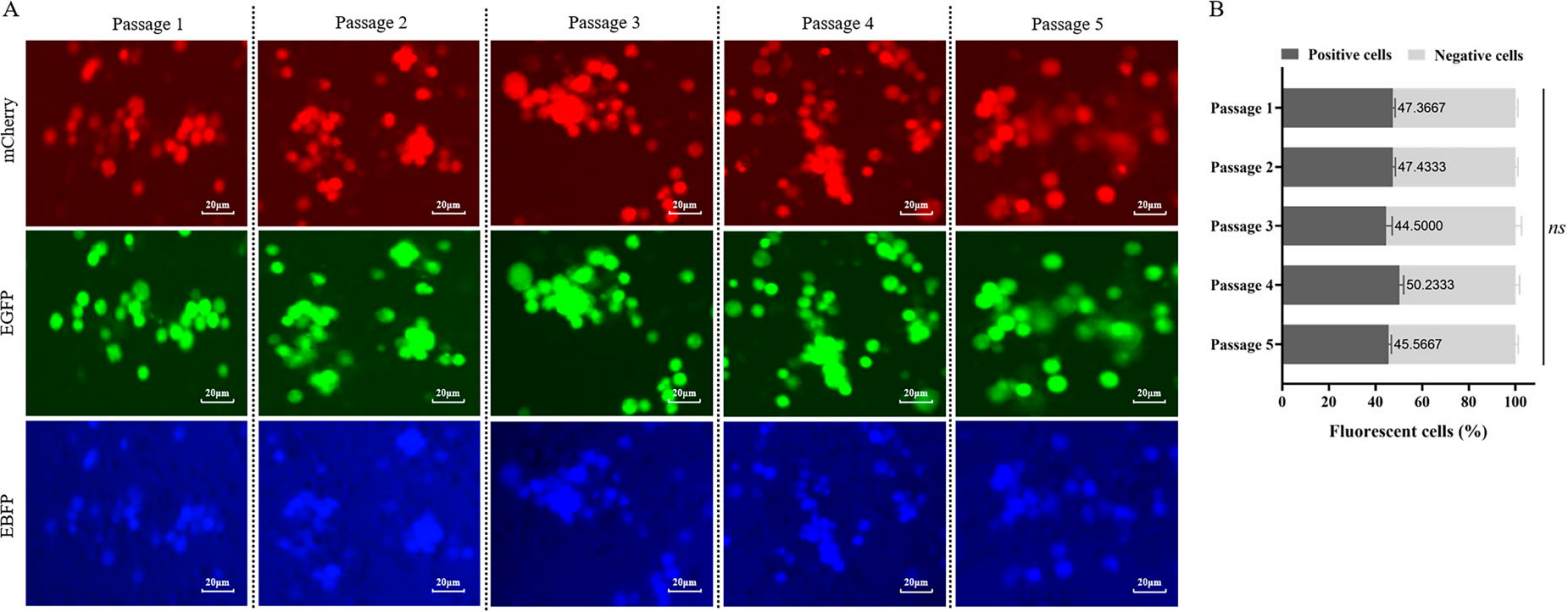
*In vitro gene stability assessment.* (A) The ASFV-GZΔI177LΔCD2vΔMGF virus was mixed with the same dose of ASFV-GZ in equal volume (10^6.0^ TCID_50_ /ml), and then serially passed in primary BMDM cells at a 2% (V/V) dose. After 72 hpi, the presence of fluorescence from 1st to 5th passage was detected under a fluorescence microscope (ECHO Global) (10×). (B) The percentage of ASFV-GZΔI177LΔCD2vΔMGF-infected cells was determined by counting at least 200 cells from three independent experiments.

The greatest concern with gene-deleted attenuated vaccines is whether the vaccine seeds might re-acquire the gene through recombination between the vaccine strain and the wild strain. A genetic stability experiment was performed by stimulating the growth of the vaccine strain in the presence of the wild strain for five passages to test whether the fluorescent reporter gene is lost. To this end, the vaccine virus was mixed with the same dose of wild strain in equal volume (10^6.0^ TCID_50_/ml), and then infected primary BMDM cells at a multiple of infection (m.o.i) of 0.01. After 72 h post-infection, the presence of fluorescence was detected under a fluorescence microscope (ECHO Global). The virus culture was harvested for four more passages in primary BMDM cells at a 2% (V/V) dose. The results are displayed in Figure 7. In the field of view of different passages, no single-fluorescence or double-fluorescence cells were observed, but green, red, and blue fluorescence coexisted (Figure 7(A)). Additionally, the percentage of ASFV-GZΔI177LΔCD2vΔMGF-infected cells in different passages was maintained at 42.7∼52.3% without significant difference (Figure 7(B)). These results indicated that the ASFV-GZΔI177LΔCD2vΔMGF virus is unlikely to recombine with the wild strains to re-acquire the deleted genes in their genome. Furthermore, we purified the virus in passage 5 by plaque purification and limiting dilution, followed by nanopore sequencing to evaluate its gene stability. The results showed that the virus in passage 5 did not re-acquire the deleted genes and no significant genetic mutation occurred in their genome.

To further investigate the genetic stability of ASFV-GZΔI177LΔCD2vΔMGF in pigs, the ASFV-GZΔI177LΔCD2vΔMGF-positive sample (kidney) was collected from the *in vivo* reversion to virulence study (the passage 5 group) for viral DNA extraction and nanopore sequencing. The results also showed that the deleted gene segments were replaced by the Pp72-EGFP, Pp72-mCherry, or Pp72-BFP expression cassette as designed, and no significant genetic mutation occurred in their genome. Hence, it is implicated that, the ASFV-GZΔI177LΔCD2vΔMGF virus exhibits good genetic stability *in vivo*.

## Discussion

Vaccination is one of the most effective methods for virus prevention. However, no commercially available ASFV vaccine limits the spread of ASFV. Several vaccine strategies, including inactivated vaccines, subunit vaccines, DNA vaccines, and viral vector vaccines, have been tried to develop safe and effective vaccines in previous studies, while the efficacy of these vaccine candidates was not satisfactory and they did not stimulate the desired immunoprotective effect on swine [4,6,21,22]. Recent studies demonstrated that several natural isolates and recombinant viruses with different virulence-related gene deletions could induce robust protective performance [10,14,23]. Although live attenuated vaccines showed great potential in inducing strong immunoprotective against virulent ASFV challenges compared to other types of vaccines, they suffered from a poor safety profile since the vaccinated virus cannot be completely cleared by the body, persistent viremia induced a chronic form of the disease or side effects, such as skin ulcer, persistent fever, viremia or joint swelling and so on [7,8,24]. In addition, virus shedding and circulating in pigs may also pose potential threats to virulence reversion. Therefore, the selection of reasonable virulence-related targets for deletion mutants is crucial to the development of ASF vaccines.

It has been reported that more than 150 viral proteins are encoded by ASFV, many of which have immunosuppressive properties, such as CD2v, MGF, 9GL, etc. [25–27]. Current research on ASFV lives attenuated vaccines focus on the rational attenuation of virulent viruses by deleting immunosuppressive genes. Among them, CD2v and MGF family gene deletion strains are mostly studied. CD2v is a structural protein that is multifunctional. It is involved in cell–cell adhesion, T-cell-mediated immune responses, virus replication, and *in vivo* dissemination and immune evasion [27,28]. In addition, CD2v protein can damage the function of lymphocytes, and the expression of this protein has some connection with ASFV spreading in domestic swine. The cytoplasmic region of CD2v protein was proposed as a new genetic marker [29]. This character could be used to analyze ASFV strains from different locations and track the virus spread. In a study, it was found that deletion of *EP402R* gene did not affect the virulence of the virus, but it significantly delays the onset of viremia and means viremia titers and the spread of the virus [30]. Therefore, CD2v is considered a strong candidate target in multiple genetic deletions and could be differentiation of vaccinated from infected animals (DIVA). There are five multi-gene families in ASFV which have been implicated in regulating the immune responses and host specificity, including MGF360, MGF110, MGF505/530, MGF300, and MGF100 groups [31–33]. Previous studies have documented that deleting the MGF genes reduce the virus replication in macrophages and deletion of MGF genes will reduce the virulence of ASFV, but exhibited prolonged viremia, which has potential safety issues [34]. In addition, the multigene family genes mainly exist in the highly variable end genomic region and have high amino acid sequence similarity. Therefore, live attenuated virus based on the deletion of MGF family genes can easily revert their virulence through self-mutation and homologous recombination. Interestingly, the deletion of the *EP402R* gene (CD2v) eliminates the risk of conversion to a virulent strain in a Chinese strain bearing a seven-gene-deleted [16]. Moreover, the recently reported *I177L* deletion of the ASFV Georgia/2007 strain (ASFV-G-ΔI177L) was friendly to 80–90 pound commercial pigs, and results in sterile immunity against the virulent strain [10]. Undoubtedly, *I177L* is a key virulence-related gene. However, Xu et al. have reported that the Chinese strain GZ201801 had undergone more variation than the Georgia strain ASFV-G ^35^. Therefore, it is necessary to carefully evaluate whether the deletion of *I177L* based on Chinese strain GZ201801 would have an excellent performance. For ASFV live attenuated vaccines, safety is still the top priority, especially for weaned piglets. As single gene-deleted live attenuated vaccines elicit a robust immunity in pigs to ASFV, additional genetic deletions may be necessary to improve their safety profile as the next-generation live attenuated vaccine.

Here, three gene-deleted ASFVs were constructed (ASFV-GZΔI177L, ASFV-GZΔI177LΔCD2v, and ASFV-GZΔI177LΔCD2vΔMGF) based on genotype II ASFV Chinese strain GZ201801, and their biological properties were analyzed. The results showed that these three gene-deleted ASFVs were able to be rescued in primary BMDM cells, but significantly reduced the replication ability of the virus. *In vivo* evaluation, weaned pigs (35-day-old piglets, 10–15 kg) inoculated intramuscularly with these three gene-deleted ASFVs survived the 28-day immunization observation period. However, pigs inoculated with ASFV-GZΔI177L or ASFV-GZΔI177LΔCD2v developed more or less adverse effect. Two out of five pigs inoculated with ASFV-GZΔI177L showed mild joint swelling at the end of immunization observation period, and all pigs infected with ASFV-GZΔI177L exhibited higher levels of viremia at 21–28 dpi as reported by Borca et al [10]. Three of five pigs inoculated with ASFV-GZΔI177LΔCD2v also developed mild joint swelling after 7–10 days post-challenge, which progressively worsened over time. Additionally, one of the pigs inoculated with ASFV-GZΔI177LΔCD2v developed a transient fever at 3–4 days post challenge. In contrast, none of the pigs inoculated with ASFV-GZΔI177LΔCD2vΔMGF exhibited the above-mentioned adverse effects during the 28 days of observation period. Most importantly, they were protected when challenge with the ASFV virulent parental strain GZ201801. Several lines of evidence suggest that ASFV-GZΔI177LΔCD2vΔMGF is a highly effective ASF vaccine candidate. First, most pigs inoculated with ASFV-GZΔI177LΔCD2vΔMGF displayed a lower viremia than that of the pigs in the ASFV-GZΔI177L- and ASFV-GZΔI177LΔCD2v-inoculated groups, and the virus can be completely expunged during the 28-day immunization period. Second, the ASFV-GZΔI177LΔCD2vΔMGF has good genetic stability, whether serially passed *in vitro* or *in vivo*. Four, no viral shedding was detected during the immunization observation period in the ASFV-GZΔI177LΔCD2vΔMGF-inoculated group. This is a desirable characteristic of a potential candidate live attenuated vaccine.

The host immune mechanisms mediating protection by attenuated strains against virulent ASFV remain unclear. In this report, we observed that all the animals that survived the inoculation developed high levels of ASFV-specific antibodies before the challenge. These results are in agreement with previously reported that animals inoculated with vaccine candidate ASFV-G-ΔI177L presenting with ASFV-specific antibodies were fully protected against lethal ASFV-G challenge [10]. However, the ASFV-specific antibodies induced by these three dene-deleted ASFVs did not have viral neutralization activity, indicating that the role of ASFV-specific antibodies may not be critical in the immune protection mediated by gene-deleted ASFVs in this study. Additionally, interferons (IFNs) are generally considered to be associated with the limited replication of attenuated strains *in vivo* [36,37]. Evidence suggests that all the animals that survived the inoculation developed extremely high levels of IFNs, especially IFN-β, which is the opposite of its parental strain ASFV-GZ. The results indicated that the surviving pigs inoculated with these three gene-deleted ASFVs are likely to be associated with increasing innate immune response.

In summary, we constructed a gene-deleted virus ASFV-GZΔI177LΔCD2vΔMGF by deleting the genes of *I177L*, *CD2v*, *MGF360-12L*, *MGF360-13L*, and *MGF360-14L* from the virulent ASFV-GZ strain. The ASFV-GZΔI177LΔCD2vΔMGF are fully attenuated in pigs, have a low risk of converting to a virulent strain, and protected pigs against challenges with the virulent parental virus. All our findings revealed that ASFV-GZΔI177LΔCD2vΔMGF is a promising attenuated vaccine candidate for ASF control. In the future, we are interested in continuing to explore more gene deletion targets based on ASFV-GZΔI177LΔCD2vΔMGF vaccine candidate to balance the safety and immunogenicity, and to further analysis the mechanism by which it exerts vaccine function.

## Materials and methods

### Biosafety statement

Animal experiments, as well as ASFV infections, were carried out in animal biosafety level 3 (ABSL-3) at Spirit Jinyu Biological Pharmaceutical Co., Limited. In addition to the Chinese National Accreditation Service for Conformity Assessment (CNAS) (license no. CNAS-BL0101) these experiments have also been approved by the Ministry of Agriculture and Rural Affairs.

### Virus and cells

As previously described [38], we have prepared the primary BMDM cells from the medullary cavities of 40-day-old piglets and cultured them in RPMI-1640 containing 10% fetal bovine serum (FBS), 1% Pen-Strep, 2 mM L-glutamine, and 10 ng/ml rpGM-CSF (R & D System, Minneapolis, MN, USA), And then, incubated at 37 °C in a humidified atmosphere with 5% CO_2_. The ASFV strain GZ201801 (abbr. ASFV-GZ) was isolated from a piglet with a severe infection in Guangdong during 2018 and propagated using primary BMDM cells as previously described [19]. Primary BMDM cells were used for virus titration, and the virus titre was calculated using the Reed-Muench method [39].

### Homologous recombination

Transfection and infection procedures were used to create ASFVs with different gene deletions generated by homologous recombination between the ASFV-GZ virus and recombination transfer vectors [17–19]. Fusing polymerase chain reaction (PCR) was used to create the transfer vectors. It contained the flanking genomic regions of the targeted genes (1.2 kb) as well as the reporter gene cassettes containing the *EGFP*, *mCherry*, or *BFP* genes which are under the control of ASFV p72 late gene promoter (Pp72) and being inserted into the sequence space between the left and the right arms to selectively remove the targeted genes as depicted in Figure 1(A). JetPEI®-macrophage transfection (Polyplus-transfection Inc., Illkirch, France) was used to transfect primary BMDM cells with 2 μg of recombinant transfer vectors and infected with ASFV-GZ at a dose of 1 m.o.i at 6 h post-transfection (hpt). The cells expressing fluorescence were picked out from the primary BMDM monolayers under the fluorescence microscope and passed into fresh primary BMDM cells. After 8–10 rounds of screened and further limiting dilution purification processes, the gene-deleted virus was purified to homogeneity. Using these constructions, we generated gene-deleted ASFVs with different gene deletions, designated as ASFV-GZΔI177L (a 111-bp deletion in the *I177L* gene: nucleotides positions 174, 600 nt and 174, 710 nt), ASFV-GZΔI177LΔCD2v (*I177L* and *CD2v* genes deleted), and ASFV-GZΔI177LΔCD2vΔMGF (including the five genes encoding the corresponding proteins of I177L, CD2v, MGF360-12L, MGF360-13L, and MGF360-14L), respectively.

### Quantitative PCR assay

GlinX Viral Nucleic Acid Extraction kit (GlinX, Shanghai, China) was used to extract genomic DNA from tissue homogenate (Tissuelyser-FEII, Shanghai, China), oral swabs, nasal swabs, anal swabs, or whole peripheral blood. qPCR was performed on a Quant Studio 5 system (Applied Biosystems, USA) following the protocol of the ASFV dtec-qPCR kit (Genetic PCR Solutions^TM^, Spain). ASFV genomic DNA copies were calculated according to a standard curve (y = 13.006-0.3017x; y: viral DNA copies/ml, x: C*t* values) derived from testing plasmids that contained the full length of the ASFV-GZ *B646L* gene.

In addition, a pair of *I177L*-based primers and probes were also designed using Primer Premier 6 software to distinguish between the gene-deleted virus and the wild-type virus (I177L-F: TGGAAAGTTAATGATCAGGGCTT; I177L-R: GGCATAATT ATCAAATG CGAAGGG; I177L-Probe: CY5-AGCCATTACCGGCAAGCTAGGATT-BHQ-1).

### Animal trials

The piglets, weighing about 10–15 kg, were procured from a local farm that adheres to the highest standards of biosecurity and hygiene. A PCR or qPCR was used to detect common porcine viruses [19]. To evaluate the virulence of the gene-deleted ASFVs among pigs, around 15 piglets were randomly divided into three groups and inoculated intramuscularly with 10^5.00^ TCID_50_ of each gene-deleted ASFV, respectively. In two weeks, the pigs were again boosted with the same dose and route of infection. By the clinical scoring system described previously [40], rectal temperatures were monitored and clinical symptoms recorded. The qPCR method was used to detect viral DNA in clinical samples of sera, heparin-anticoagulated whole blood, oral swabs, nasal swabs, and anal swabs collected after 0, 3, 5, 7, 9, 14, 21, and 28- days post-infection. To assess the protective efficacy of these gene-deleted ASFVs, all pigs were intramuscularly challenged with 10^4.00^ TCID_50_ of lethal ASFV-GZ virus post 28 days after inoculation. As a mock-vaccinated control group, five pigs were challenged. Clinical signs associated with the disease were recorded as described earlier [19]. The pigs were necropsied following clinical assessments. We scored and collected samples of tonsils, liver, spleen, kidneys, heart, lungs, thymus, and lymph nodes for qPCR detection as previously described [20].

### Safety evaluation in pigs

In order to investigate ASFV-GZΔI177LΔCD2vΔMGF replication in pigs, about 8 commercial domestic pigs (2-month-old) were inoculated with a high dose of the virus (10^7.00^ TCID_50_). Subsequently, one pig was euthanized respectively on days 3, 5, 7, 10, 14, 30, 45, and 60 p.i., and clinical samples including the blood, heart, liver, spleen, lung, kidney, tonsil, thymus as well as eight lymph nodes (lumbar lymph nodes, submaxillary lymph nodes, inguinal lymph nodes, gastro-hepatic lymph nodes, hilar lymph nodes, groin lymph nodes, mediastinal lymph nodes, and mesenteric lymph nodes, etc) were collected for viral DNA detection. To ensure the safety of ASFV-GZΔI177LΔCD2vΔMGF in pigs, we conducted an additional safety evaluation by following the guidelines for emergency evaluation of ASF vaccines and the Guidelines for Genetically Modified Organisms in China. In brief, groups of three commercial piglets were inoculated intramuscularly with 10^7.00^ TCID_50_ of ASFV-GZΔI177LΔCD2vΔMGF virus, out of which two are euthanized at 3 dpi, in which their organs and tissues were collected for the viral DNA detection, while the rest one is observed for 21 days. Finally, we have homogenized the DNA-positive samples and inoculated them into pigs for four more passages in pigs.

### *In vitro* and *in vivo* gene stability assessment

We evaluated the genetic stability of ASFV-GZΔI177LΔCD2vΔMGF in primary BMDM cells. In brief, both the ASFV-GZΔI177LΔCD2vΔMGF and parental ASFV-GZ viruses were diluted to 10^6.00^ TCID_50_ / ml in DMEM medium and mixed in equal volumes. Monolayer primary BMDM cells were grown in 12-well plates (5–7 × 10^6.00^ cells/ml, 2 ml/well) and infected at a multiple of infection (m.o.i) of 0.01 with the mixed virus. For five consecutive passages, the virus solution was harvested at 72 h post-infection (hpi) and infected the primary BMDM cells at a 2% (v/v) dose. An ECHO Global fluorescence microscope was utilized to observe fluorescent cells and the presence of fluorescence was used as an indicator to determine if ASFV-GZΔI177LΔCD2vΔMGF could recombine with the wild-type ASFV to acquire the deleted genes.

Furthermore, the ASFV-GZΔI177LΔCD2vΔMGF-positive samples of passage 5 group were collected from the safety evaluation study for viral DNA extraction and nanopore sequencing (Oxford Nanopore Technologies, Oxford, UK) to evaluate their genetic stability *in vivo*.

### Indirect ELISA

Cytokines in the sera were detected using ELISA Kit (Solarbio) according to the manufacturer’s instructions. The detected cytokine types and the corresponding minimum detectable doses of IL-6, TNF-α, IFN-α, IFN-β and IFN-γ were 3, 5, 3, 7 and 6 pg/ml, respectively. The anti-ASFV antibodies in the sera were detected using an African Swine Fever Competition ELISA Kits (IDVet Innovative Diagnostics Louis Pasteur, Grabels, France) and also calculated the competition percentage (S/N %). As a general rule, samples exhibiting an S/N % ≤ 40% were considered positive, 40–50% were considered doubtful, and ≥ 50% were considered negative.

### Virus neutralization test

Virus neutralization test were performed on the serum samples collected at 28 dpi from pigs vaccinated with these three gene-deleted ASFVs. The serum samples were diluted by two-fold serial dilution with RPMI-1640 medium, and then mixed with the same volume of ASFV-GZΔI177L (100 TCID_50_). All samples were incubated at 37°C for 2 h and then added to the primary BMDM cells in 96-well plates (2 × 10^5.00^ cells/well) and cultured at 37°C under 5% CO_2_. The fluorescent cells were observed under an ECHO Global fluorescence microscope from 24 to 96 hpi and the presence of fluorescence was used as an indicator to determine if the sera could neutralize the ASFV.

### Statistical analysis

We have used GraphPad Prism software (GraphPad Software Inc., La Jolla, USA) for performing statistical calculations. The data are presented as means ± the standard deviations (SD). Statistical analyses were performed using unpaired, two-tailed Student *t*-test. A *p*-value less than 0.05 is considered statistically significant.

## Data and materials availability

The Nanopore raw sequencing date (FASTQ format) reported in this study have been deposited in the NCBI Sequence Read Archive (SRA Accession no. PRJNA866680). Other data from this study are available in the main text or supplementary materials.
